# Supplementary material for: One-Step Green Synthesis of Water-Soluble Fluorescent Carbon Dots and Its Application in the Detection of Cu2+
Source: Nanomaterials (Basel). 2022 Mar 14;12(6):958. doi: 10.3390/nano12060958 (PMC8952276; doi:10.3390/nano12060958)
Supplement: Supplementary file 1 [file nanomaterials-12-00958-s001.zip › nanomaterials-1602421-supplementary.pdf]

nanomaterials

One-Step Green Synthesis of Water-Soluble Fluorescent Carbon Dots and Its Application in the Detection of Cu^{2+}

Saheed O. Sanni [1,2,*], Theo H.G. Moundzounga [2], Ekemena O. Oseghe [3], Nils H. Haneklaus [4,5], Elvera L. Viljoen [2] and Hendrik G. Brink [1,*]

S1 Quantum yield measurement

The quantum yield of synthesized PC-CDs at an optimized excitation wavelength of 430 nm was determined by using rhodamine B (QY is 31%, dissolved in water with refractive index of 1.33) as our reference standard, while the PC-CDs were dissolved into ultrapure water (refractive index of 1.44) and calculated according to the equation below:

$$\phi_{PC-CDs} = \phi_R \cdot \frac{I_{PC-CDs}}{I_R} \cdot \frac{A_R}{A_{PC-CDs}} \cdot \frac{\eta^2_{PC-CDs}}{\eta^2_R} \quad (1)$$

where Ø is the quantum yield, I is the measured integrated emission intensity, η is the refractive index of solvent employed, and A is the optical density. The subscript R represents the reference rhodamine B with known quantum yield.

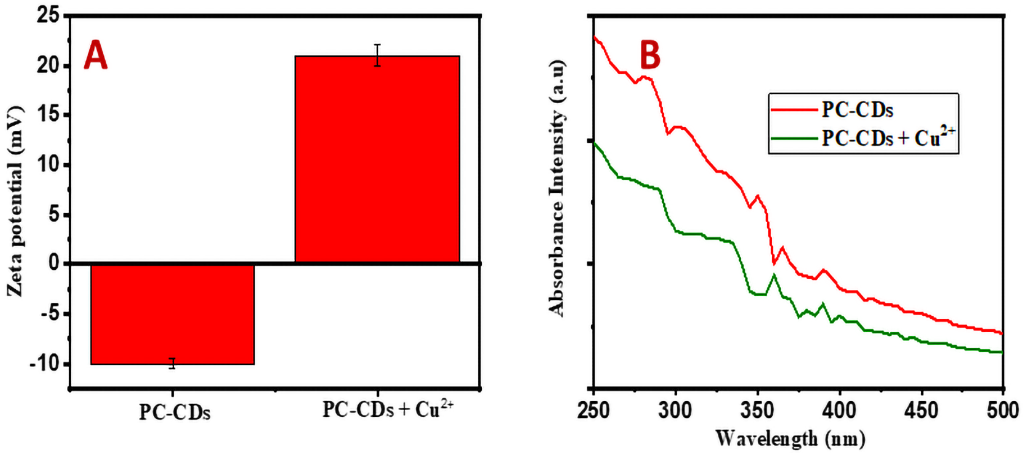

Figure S1. (**A**) Zeta potential of PC-CDs alone, and PC-CDs with the addition of Cu^{2+} metal ions, and (**B**) UV-Visible spectra of PC-CDs alone, and after addition of Cu^{2+} ions.

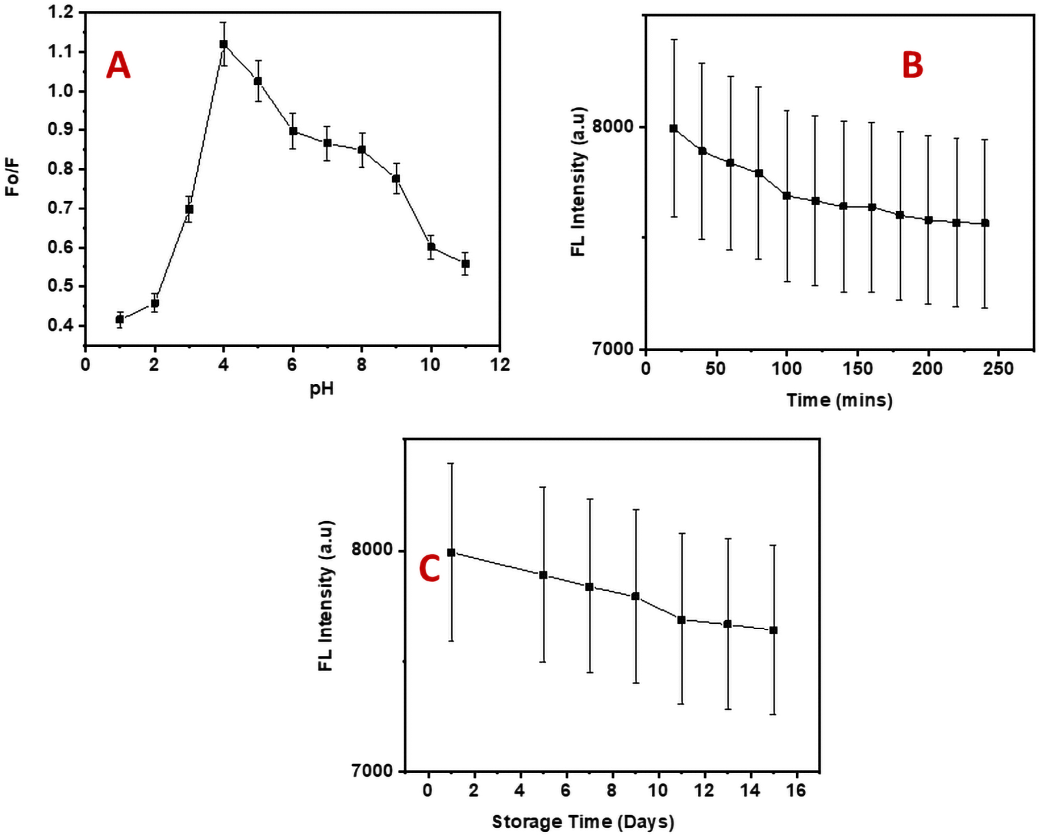

Figure S2. (**A**) pH effect on PL intensity of synthesized PC-CDs (**B**) Photostability of PC-CDs under UV lamp (365 nm) for 2 h, and (**C**) under normal sunlight storage.

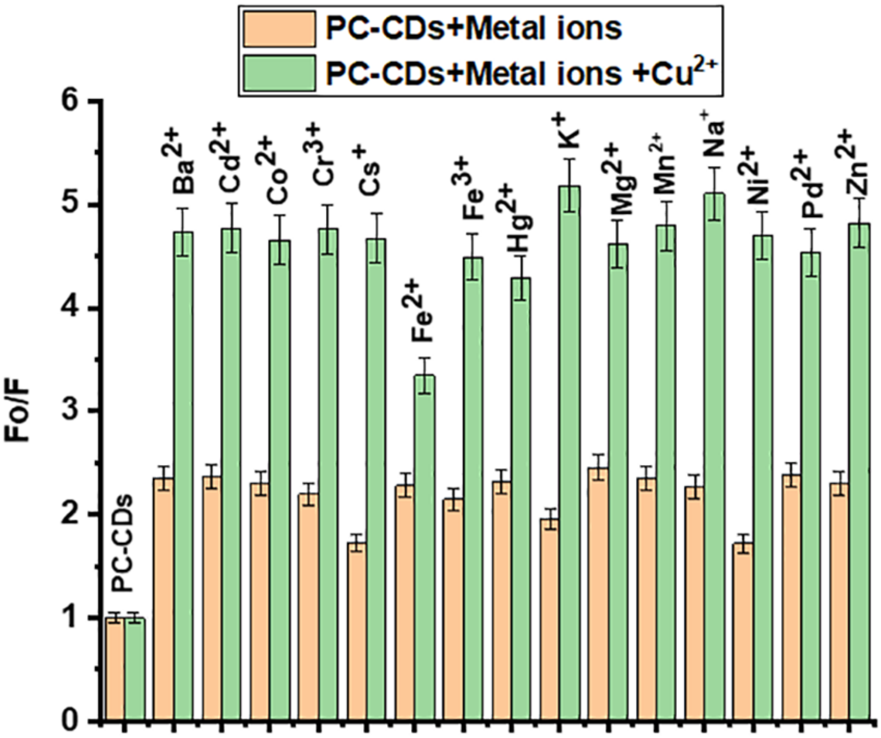

Figure S3. PL intensities synthesized PC-CDs in the presence of interfering metal ions (brown bar), and the mixture solution of Cu^{2+} with interfering metal ions (green bar).

Table S1. Detection of Cu^{2+} in real waste water effluent.

Sample	Added (μg/mL)	Found (μg/mL)	Recovery (%)
	0.5	0.42	84.14
PC-CDs	2	2.02	101.07
	3	3.17	105.59
	4	3.92	98.13
	6	5.97	99.45
